# Grasping and Pointing in Visual Periphery: Consistent Impairments in Visual form Agnosic Patient DF

**DOI:** 10.1068/ig11

**Published:** 2013-10-01

**Authors:** C Hesse, T Schenk

**Affiliations:** University of Aberdeen, UK; Universität Erlangen-Nürnberg, Germany

## Abstract

In a recent study, we observed that the visuomotor performance of visual form agnosic patient DF is significantly impaired when targets are presented in visual periphery (Hesse, Ball, & Schenk, 2012). Based on the observation that DF's reaching and grasping behaviour is compromised in visual periphery, we argued that her performance cannot be described as being complimentary to that of patients with optic ataxia (as suggested previously). However, there are two possible explanations for our findings. Firstly, DF's visuomotor deficit might indicate that an intact ventral stream is indispensable for the programming and execution of certain visuomotor tasks. Secondly, as DF also shows a small lesion in the left posterior parietal cortex, her failure to perform accurate movements in visual periphery might indicate that she also suffers from an optic ataxia. In a follow-up study, we therefore investigated whether patient DF shows a typical “hand-effect” or “field-effect”, as commonly observed for optic ataxia patients with unilateral lesions. That is, we systematically tested her visuomotor performance when pointing with the left or the right hand into the left or right visual field. Results show that DF's visuomotor deficit occurs independently of the visual field in which the stimuli were presented, as well as the hand with which movements were performed. These findings do not support the hypothesis that unilateral optic ataxia is responsible for the visuomotor deficits in patient DF. Alternatively, we suggest that damage to ventral stream areas can lead to profound visuomotor deficits in specific situations.

